# Ultrasound technology in fish proteins processing: Innovations in extraction and structure–function dynamics

**DOI:** 10.1016/j.ultsonch.2025.107503

**Published:** 2025-08-08

**Authors:** Samaneh Pezeshk, Mehdi Abdollahi

**Affiliations:** a Department of Marine Biology–Marine Biotechnology, Faculty of Marine Sciences, Tarbiat Modares University, P.O. Box 46414-356, Noor, Iran; b Department of Life Sciences–Food and Nutrition Science, Chalmers University of Technology, SE 412 96 Gothenburg, Sweden

**Keywords:** Marine proteins, Protein functionality, Sonication, Green processing, Side stream valorization, Sustainable food processing, Protein structuring

## Abstract

Fish proteins are known for their high nutritional value and excellent functional qualities, which is why they could be susceptible to wide application in the food and health industries. However, their structural complexities and functional constraints during processing may limit their usefulness. In recent years, ultrasound technology has received significant interest as an effective method for modifying fish protein’s structure through the cavitation effect to improve their functionality. This review highlights current advances in ultrasound-assisted fish protein extraction, with an emphasis on structural modification and functional characteristics augmentation. The paper thoroughly assesses existing scientific knowledge and discusses potential research directions. Overall, ultrasound technology has the potential to be a successful assistant technique for improving fish protein extraction efficiency, along with benefits such as faster processing and lower resource use. Ultrasound can significantly improve the functionality and technological applicability of fish proteins by reducing aggregation, modifying secondary and tertiary structures in aqueous systems, and enhancing molecular flexibility and interfacial activity. However, it should be highlighted that achieving these great synergistic effects necessitates optimizing processing settings based on the specific ultrasound equipment employed, the fish species under study, and the type of protein. Future studies should focus on using ultrasound to carefully tune the structure of fish proteins for engineering and tailoring their functionality to boost their compatibility for complicated food processing techniques such as high moisture extrusion, 3D printing and for the development of hybrid foods in combination with emerging protein sources.

## Graphical abstract

**Figure:**
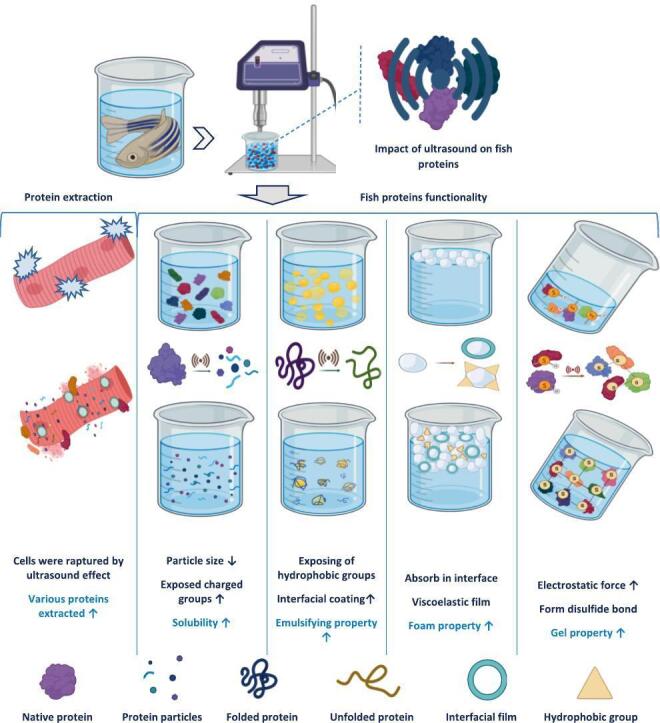

## 1 Introduction

Proteins are essential not only for human nutrition and physiology but also as versatile building blocks in the development of innovative food products, owing to their unique techno-functional properties [1]. Gel formation, foaming ability, emulsifying capacity, solubility, and water-holding capacity are key techno-functional properties that make proteins valuable in food applications [2]. However, most proteins in their native state do not exhibit all the desired functionalities, necessitating structural modification to improve these properties to broaden their application potential [3]. During food processing, proteins may also undergo various transformations, such as unfolding, coagulation, aggregation, and exposure of hydrophobic groups, leading to changes in their fundamental structure and, consequently, their functionality. [4]. By carefully selecting processing techniques and controlling their conditions, the type and extent of protein structural changes can be tuned, opening new opportunities to improve and tailor protein functionality for specific applications.

Proteins can be sourced from a wide range of plant and animal origins. Among them, aquatic proteins, particularly those from fish, have gained increasing consumer interest due to their excellent essential amino acid profile, high biological value, and alignment with the growing demand for healthier dietary choices [5]. In addition, compared to proteins obtained from terrestrial organisms, consumption of aquatic resources is less restricted due to a lower frequency of diseases and fewer religious constraints [6]. However, focusing on fish fillet as the main protein source has resulted in generating huge amounts of by-products, including heads, fins, spines, viscera, and skin during the industrial processing of fish, reaching 50–70 % of the overall weight of the fish. These by-products frequently end up in low-value uses like animal feed, fish fertilizer, and fish silage or are even discarded, which causes environmental pollution. These by-products constitute a substantial untapped resource and are abundant in important nutritional elements such as proteins, vitamins, vital minerals, and polyunsaturated fatty acids [7]. In addition to their substantial nutritional value, fish proteins are widely used in the food sector due to their excellent functional properties [8]. Their unique functional qualities, such as gelling, foaming, stabilizing, thickening, and emulsifying, also contribute to the diversity of food items [9].

Increasing attention has been given to the development of milder processing techniques due to the high sensitivity of fish proteins to denaturation, oxidation, and thermal degradation under conventional convective extraction conditions. Additionally, researchers are prioritizing the development of environmentally sustainable extraction processes or green technologies due to the growing environmental concerns [10]. In response, a wide variety of non-thermal and green technologies such as ultrasonication, pulsed electric fields, UV light, high-pressure processing, and cold plasma treatment have been targeted for the extraction, processing and modification of fish proteins. Unlike conventional methods, these approaches offer cleaner, more sustainable, and energy-efficient alternatives that preserve the nutritional integrity and functional properties of proteins [9,11]. Among these emerging methods, ultrasound has garnered significant scientific interest due to its acoustic cavitation effects, environmental compatibility, and ability to modify proteins without the use of heat or harsh chemicals. In addition, Ultrasound-assisted extraction of fish proteins typically yields higher protein recovery while significantly reducing required solvents and extraction time [12].

Cavitation is the primary mechanism by which ultrasound exerts its effects on liquid systems and is widely regarded as the most significant outcome of ultrasound treatment. This phenomenon enhances both chemical and physical processes by promoting efficient mass transfer, generating localized hot spots, forming highly reactive free radicals, and creating turbulent microenvironments [10,13]. Compared to conventional solvent-based extraction methods, ultrasound is considered a safer and more environmentally friendly approach, with broad applicability in protein modification, enzyme activation or inactivation, and the separation, mixing, and extraction of liquid-based food matrices.

Ultrasound, in particular, has shown great promise in improving the functional properties of proteins, such as solubility, gelation, and emulsification, by inducing structural changes [14]. These modifications are primarily driven by cavitation, shear forces, turbulence, and localized heating if the system is not adequately cooled during ultrasound treatment [15]. Under specific ultrasound conditions, the generation of reactive radical species in water may also lead to further alterations in protein function. While ultrasound can cause changes to the secondary, tertiary, and quaternary structures of proteins, the primary structure generally remains intact, preserving the protein’s identity while enhancing its functional versatility [3]. However, due to the sensitive nature of fish proteins, careful optimization of ultrasound processing conditions is essential to prevent potential adverse effects, such as protein aggregation, which can occur under suboptimal ultrasonication settings and compromise protein functional properties [10].

**Fig. 1:**
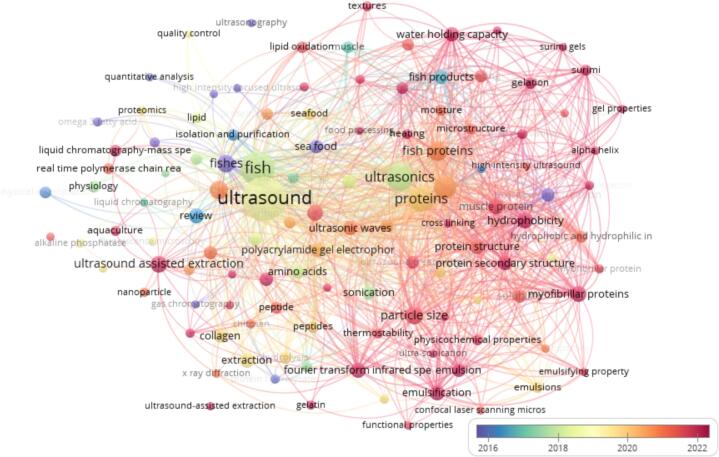
Visualization of the current status of the effects of ultrasound for enhancing extraction and functional properties of fish proteins.

Ultrasound-assisted protein modification has been the subject of much research, which has examined its possibilities for a variety of protein sources. To the best of the author's knowledge, however, no thorough review particularly discusses the use of ultrasound for the modification of fish protein’s functionality. Therefore, using VOSviewer software for information analysis visualization, we conducted a literature search in the Scopus database for articles about ultrasound, fish protein, and functional properties published between 2015 and 2025 in order to assess the current state of research in this field (see Fig. 1). These findings suggest that a growing number of scholars are contributing significantly to this discipline, which is producing high-caliber publications. This suggests that there is a large and established research group focused on using ultrasound to modify fish proteins, albeit some researchers may need to strengthen their teamwork. Many linked research centers are revealed by looking at keywords associated with the ultrasound treatment of fish proteins. Frequently used terms such as technique, ultrasound, extraction, structure, solubility, emulsion, water holding capacity (WHC), gel strength, hydrolysis, and bioactive compound highlighted the importance of examining extraction methods and altering the characteristics of fish proteins. As a result, the advantages of ultrasound treatments are becoming more widely known, particularly about their ability to alter the structure of fish proteins. Better functional and bioactive qualities are widely reported, and since 2020, they have emerged as a new area of interest for this field of study. Despite the increasing number of publications on ultrasound-assisted extraction and modification of fish proteins, there remains a significant gap in the literature. To date, no comprehensive review has synthesized the growing body of research with a specific focus on how ultrasound influences the functional properties of fish proteins. Existing studies are often fragmented, addressing isolated effects or specific species or biomass, without providing a holistic understanding of underlying mechanisms, optimized processing parameters, or comparative outcomes across different conditions. A systematic review is therefore essential to consolidate current knowledge, identify methodological inconsistencies, and highlight areas requiring deeper investigation, such as protein structure–function relationships and long-term impacts on food quality. Moreover, there is a critical need to define the full potential of ultrasound as a tool for tailoring fish protein functionality. By precisely modifying protein structures, ultrasound can unlock new applications in food formulation, particularly within the context of the emerging future food system that demands sustainable, functional, and nutritionally rich ingredients.

Therefore, the purpose of this review is to investigate the processes and outcomes of ultrasound in the process of extracting protein from fish sources, as well as the impact of ultrasound on protein modification. With an emphasis on proteins originating from fish bio-waste, it will also investigate the ensuing structural modifications, techno-functional characteristics, and physicochemical changes in fish proteins. This review aims to provide a comprehensive understanding of how ultrasound can be strategically employed to enhance the value and functionality of fish proteins for diverse food applications.

## 2 Fish proteins

**Fig. 2:**
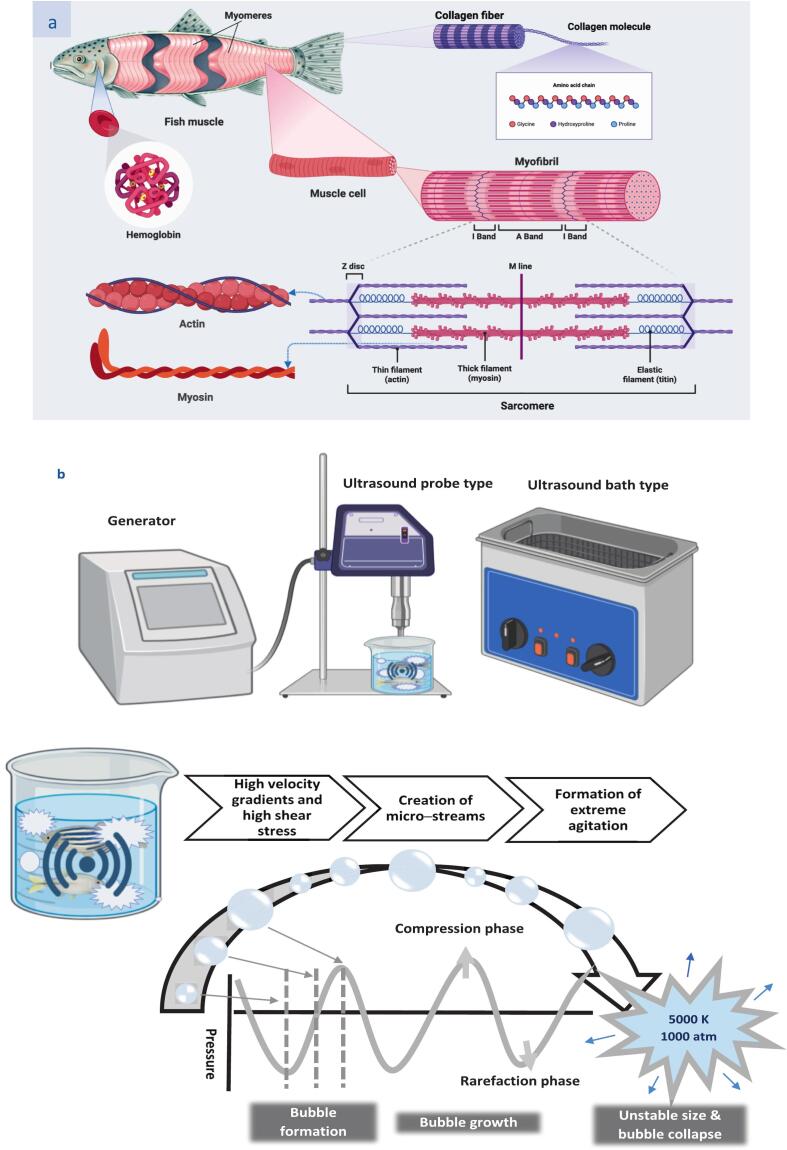
A: Overview of the main types of fish proteins; b: Schematic diagrams of acoustic cavitation induced by ultrasound and its mechanisms.

Fish contains a variety of proteins, including salt-soluble myofibrillar proteins, water-soluble sarcoplasmic proteins, and insoluble stromal proteins (See Fig. 2a). Typically, myofibrillar proteins include actin, myosin, actomyosin, and tropomyosin, while sarcoplasmic proteins are made up of heme proteins and enzymes that are involved in myoglobin and muscle metabolism. The main component of stromal proteins is collagen, which is made up of three alpha polypeptide chains that form a triple helix [16]. Myofibrillar and sarcoplasmic proteins are primarily found in fish muscle tissue, while the by-products are high in collagen [17]. The process of partial hydrolysis of collagen results in a mixture of polypeptide chains, which breaks down different intra- and intermolecular covalent bonds. As a result of its distinct chemical and physical characteristics, gelatin is widely used in a variety of industries, such as food, pharmaceuticals, and photography [18].

Furthermore, fish protein isolate and protein hydrolysate are the main subjects of considerable studies on protein extraction from fish by-products. A variety of techniques are used, producing distinct functional properties such as foaming, emulsification, and gelling. In addition to their noteworthy functional qualities, these substances have a number of therapeutic advantages, including antimicrobial effects, blood pressure reduction, immune system stimulation, renin activity reduction, anti-cancer, and anti-diabetic qualities [19].

## 3 Ultrasound technology

Ultrasound is sound waves with a frequency above the audible threshold of human hearing (>20 kHz). It has a variety of applications in various industries, such as medicine and food industry [20]. In ultrasound technology, there are key parameters that play a decisive role in the effectiveness of this method. Power density and power determine the amount of total energy transferred to the medium, while intensity and amplitude affect the degree of cavitation. Frequency affects how bubbles form and collapse and Pulse mode affects how energy is distributed in the process. The geometry and mode of application of the device, such as a probe system or ultrasound bath, also play a role in how sound waves are transmitted to the medium [21].

Low-frequency (high-intensity) ultrasound and high-frequency (low-intensity) ultrasound are the two categories of ultrasound based on intensity, and each has a distinct function. Frequencies surpassing 1 MHz are mainly used in medical and industrial imaging purposes while frequencies falling within the range of 20 kHz–100 kHz with high intensities (10–1000 W/cm2) are known as high-power ultrasound and are used in processes such as extraction, cell disruption, and improved mass transfer in the food industry [22]. As a physical modification technique, ultrasound processing technology offers a number of advantages, such as safety, reduced nutrient loss, reduced by-product generation, enhanced operating efficiency, and environmental sustainability [23]. As shown in Fig. 2b, ultrasound can produce acoustic cavitation effects, which are defined by the cycle of expansion and contraction of pre-existing microbubbles that eventually collapse within the liquid when exposed to an ultrasound field [24]. Its remarkable ability is due to the synchronization of many forces accompanying cavitation, acoustic streaming, thermal effects, and mechanical shear. When acoustic waves pass through materials, they cause cycles of compression and expansion. Cavitation bubbles can form when the acoustic wave is strong enough and the medium is fluid, as the negative pressure during the expansion phase exceeds the fluid’s capacity to expand. These bubbles then absorb acoustic energy, causing them to grow in size [25]. These bubbles collapse violently upon reaching a threshold size, releasing acoustic energy manifested as turbulence, shear forces, and intense localized cavitation. During bubble collapse, localized regions experience extremely high pressures, temperatures, and shear rates for brief periods. These extreme conditions can cause physical changes in materials and generate free radicals that initiate chemical reactions. Consequently, these pressures can alter the characteristics of proteins [26].

## 4 Ultrasound-assisted fish protein extraction

Ultrasonication has emerged as an effective alternative to conventional extraction methods for food due to its rapid processing and operational simplicity. While cavitation can dislodge particles and expose protein cores, its effects are not inherently non-destructive [27]. Protein extraction is primarily enhanced through cavitation-driven mechanisms that promote mass transfer and cellular disruption [17]. These effects enhance solvent penetration into damaged cells and improve the recovery of intracellular compounds and cell wall-associated molecules. During protein extraction, ultrasonication often facilitates the breaking of intra- and intermolecular covalent cross-links, contributing to greater protein solubilization [20]. Ultrasound technology is particularly effective for moist or high-moisture biomass materials, such as fish tissues, where traditional drying steps can be avoided [13]. Despite its benefits, prolonged ultrasonication can intensify cavitation effects, resulting in high temperatures, increased pressures, and large shear forces in the medium. These conditions may disrupt van der Waals forces and hydrogen bonds in polypeptide chains, potentially causing protein or enzyme denaturation [28]. To mitigate these risks, samples are commonly maintained at approximately 4 °C using external cooling systems, along with intermittent pulsing cycles (e.g., 2–20 s on/off) to reduce heat buildup and preserve protein integrity.

The efficiency of ultrasound-assisted protein extraction is determined by the interaction of system parameters and solvent properties unique to the target molecule. Ultrasound power density, intensity, frequency, duration, temperature, and solid-to-solvent ratio are all important considerations for extraction efficiency [29]. High-intensity ultrasound (10 to 1000 W/cm^2^) has a higher energy density (1–10 W/mL) and can increase protein yields up to an acceptable threshold. Beyond this limit, yield may drop due to the excessive collapse of cavitation bubbles, which can cause turbulence that impede extraction. Furthermore, the duration of ultrasound exposure is important, since short bursts of high-intensity ultrasonication are desirable for increasing efficiency while reducing denaturation risk [30].

Temperature control is crucial in ultrasound-assisted extraction of fish proteins, as excessive heat can reduce cavitation efficiency and cause protein denaturation. Since fish proteins, particularly heat-sensitive types like collagen and sarcoplasmic proteins, are prone to structural changes at high temperatures, maintaining low temperatures helps preserve protein quality. Strategies such as pulsed ultrasound operation and the use of cold water baths during extraction are effective in minimizing heat buildup and protecting protein functionality [10]. Low-frequency, high-intensity ultrasound is very useful because it generates large shear forces and mechanical stresses, which improve extraction. In contrast, high-frequency, low-power ultrasound often causes the production of reactive radicals rather than assisting extraction [31]. Most studies show that lower consistent frequencies result in larger cavitation bubbles and more effective implosion currents. However, when frequency increases, the time available for bubble formation decreases, reducing their implosive potency and potentially impeding mass transfer [23]. Water is frequently the preferred solvent for protein extraction, with pH changes made to improve solubility [10]. The particle size and solid-to-solvent ratio have a major impact on extraction efficiency. Smaller protein molecules with larger surface areas have improved solvent diffusion and enhanced cavitation effects, resulting in higher yields than bigger particles. According to research, larger solid-to-solvent ratios enhance protein yields until an ideal equilibrium is attained, at which point yields may begin to drop [32]. Low solid-to-solvent ratios cause high viscosity, which prevents cavitation due to stronger cohesive forces, but higher ratios increase cavitation by lowering viscosity and concentration [21]. Finally, the correct position of the sonotrode tip is critical for successful extraction. In many setups, placing the tip centrally in the extraction medium at a depth of 2–3 cm can improve acoustic energy distribution and cavitation effects. The design of the sonoreactor is also important; for instance, a narrow, flat-bottomed cylindrical shape is advised for homogeneous energy distribution and increased cavitational shear forces [20]. Despite this, research into the exact configurations of sonotrode tips and sonoreactor shapes that influence ultrasound extraction of macromolecules is sparse, indicating a viable area for investigation.

Fish by-products are an excellent alternative source of dietary proteins that can support human nutrition, as they are rich in high-quality proteins and constitute a substantial portion of the raw material output from seafood processing industries [17]. Efficient protein extraction from these by-products not only helps in minimizing spoilage and oxidation of aquatic resources but also preserves their valuable functional properties. Traditional extraction techniques commonly employed include salt solubilization, aqueous extraction, organic solvent extraction, enzymatic hydrolysis, alkali solubilization followed by acid precipitation (so-called pH-shift method). Each method presents unique advantages and limitations: for instance, salt extraction helps preserve the native protein structure but results in low yield and purity. Water-based methods are limited to water-soluble proteins, while organic solvents may induce protein denaturation, requiring cold processing to minimize damage. Enzymatic hydrolysis is a mild and highly effective method, yielding easily digestible peptides, but its high cost and long processing time and lack of independent product-forming capacity remain significant barriers [33]. Ultrasound-assisted extraction is gaining attention as a sustainable, efficient, and non-destructive alternative. This technique enhances protein recovery while reducing solvent use and processing time, making it increasingly popular for extracting proteins from a wide range of aquatic sources [17]. Several studies have evaluated the effects of ultrasound on protein yield from fish by-products. Table 1 summarizes relevant literature highlighting the impact of ultrasound-assisted extraction on the yield of fish proteins.

**Table 1:** A summary of ultrasound conditions and its combination with other methods used for the extraction of fish proteins, discovered optimal conditions and their effect on protein yield.

Source (protein type/fish part)	Method	Ultrasound conditions	Optimal conditions	Extraction yield	References
Myofibrillar Proteins/tilapia fillet	pH-Shift + Ultrasound	pH-Shift: 10.5, 11, 11.5 + Frequency (kHz):20; amplitude: 75 %; Power (W): 900; Time (min):3; Pulse on/off (s):3/2	pH 10.5 + Two Ultrasound treatments	Extraction of protein improved from 54.6 % to 62.6 % (with two ultrasound treatments) ↑	[34]
Myofibrillar Proteins/ whole mackerel	Sequential acid/alkalineisoelectric solubilization precipitation (ISP) extraction + Ultrasound	Acid-alkaline or alkaline-acid process + Frequency (kHz):20; amplitude: 20 % and 60 %; Power (W): 750; Time (min):10; Pulse on/off (s):5/5 and US Bath 1 h; Frequency (kHz):40	Alkaline-acid + Amplitude: 60 %	Extraction of protein improved from 83.3 % to 97.3 % (alkaline-acid with ultrasound treatments) ↑	[64]
Collagen/yellowfin tuna skin	Pepsin + Ultrasound	Frequency (kHz):20; Power (W): 400; Time (min): 5, 10, 15, and 20; Pulse on/off (s):2/3	Ultrasound for 20 min	Extraction of collagen improved from 20.6 % to 34 % ↑	[39]
Collagen/yellowfin tuna skin	Pepsinogen + Ultrasound	Frequency (kHz):20; Power (W): 400; Time (min): 5, 10, 15, and 20; Pulse on/off (s):2/3	Ultrasound for 15 min	Extraction of collagen improved from 18.5 % to 23.8 % ↑	[38]
Collagen/ Sharpnose stingray skin	Acid and pepsin + Ultrasound	Frequency (kHz): 20; Power (W): 750; Time (min): 30; Pulse on/off (s): 5/5	Ultrasound-assisted acid	Extraction of collagen improved in UASC from 20.48 % to 48.37 %↑ and in UPSC from 34.84 % to 56.65 %↑	[36]
Collagen/Seabass skins	Acid + Ultrasound	Frequency (kHz): 20; Power (W): 750; Time (min): 10, 20, and 30; Amplitude: 20, 50, and 80 %; Pulse on/off (s): 5/5	Ultrasound in Amplitude 80 %	Extraction of collagen improved from 20 % in 24 h to 20 % in 1.5 h	[65]
Collagens/ Golden carp Skin	Acid and pepsin + Ultrasound	Frequency (kHz): 20; Power (W): 750; Time (min): 10, 20, and 30; Amplitude: 20, 50, and 80 %; Pulse on/off (s): 5/5	Ultrasound in Amplitude 80 % for 30 min assisted pepsin	Extraction of collagen improved in UASC by 81.53 % ↑ and in UPSC by 180 % ↑ under optimal conditions.	[35]
Myofibrillar Proteins /rainbow troutby-products	pH-Shift + Ultrasound	Frequency (kHz): 20; Power (W): 100 and 400; intensity (W/cm2) 88 and 353; Time (min): 5, 10and 20; Pulse on/off (s): 2/3	pH 11.5 + Ultrasound (100 W, 20 min)	Extraction of protein improved from 47 % to 65.1 % ↑	[10]
Collagen/tunby-product	Acid + Ultrasound	Frequency (kHz): 20; Power (W): 300; Time (min): 5, 10, 15, 20 and 25; Pulse on/off (s): 3/2	Ultrasound for 25 min	Extraction of collagen improved from 21.49 % to 57.06 % ↑	[17]
Collagen/Clown featherback skin	Acid + Ultrasound	Frequency (kHz): 20; Power (W): 750; Amplitude: 20, 40, 60 and 80 %; Time (min): 10, 20 and 30; Pulse on/off (s): 5/5	Ultrasound in Amplitude 80 % for 30 min	Extraction of collagen improved from 23.46 % to 57.35 % ↑	[40]
Myofibrillar Proteins /salmon headand herring frame	pH-Shift + Ultrasound	Frequency (kHz):20; Power (W): 250 or 500; Time (min):20; Pulse on/off (s):10/40	For herring frame at ultrasound 250 W, for salmon head (ns)	Ultrasound-assisted protein extraction increased from herring frames at a lower water ratio of 1:3	[33]
Protamine*/Coregonus peled*	Ultrasound-assisted acid extraction	Frequency (kHz):20; Power density (W/cm2): 9.10; Time (min): 10, 20, 30, 40, and 50	Ultrasound for 30 min	Extraction of protamine improved from 3.4 % to 6.8 % ↑	[41]
Collagen from Albacore	Ultrasound	Frequency (kHz): 20; Power (W): 150, 300, 450, and 600; Time (min): 30; Pulse on/off (s): 2/2	Ultrasound in 600 W	Extraction of collagen improved from 69.26 % to 75.18 % ↑	[4]
Protein concentrate from theflesh of snakehead fish	Ultrasound	Power (W): 100, 200, 300, and 400; Time (min): 10, 15, 20, 25 and 30	Ultrasound in 600 W for 25 min	Extraction of protein concentrate improved from 58.64 % to 73.17 % ↑	[66]
Collagen from fish scales	Ultrasound + Natural deep eutectic solvents	Frequency (kHz): 20; Power (W): 450; Time (min): 20; Pulse on/off (s): 5/5	Ultrasound in 450 W for 20 min	Extraction of collagen improved from 68.2 % to 88.4 % ↑	[60]
Protein from grass carp	Ultrasound	Frequency (kHz): 35 and 130; Power (W): 52 and 118; Time (min): 20, 30, and 40; Pulse on/off (s): 5/5	Ultrasound in Frequency of 30 kHz for 30 and 40 min	Extraction of protein improved from 3.6 % to 16.86 % ↑	[67]
Gelatin from Hilsha Scale	Ultrasound	Frequency (kHz): 20 and 130; Power (W): 200; Time (min): 60; Pulse on/off (s): 5/2	Ultrasound in 200 W for 60 min	Extraction of gelatin improved from 20.06 % to 34.49 % ↑	[9]

Tian et al. [34] explored how the ultrasound-assisted alkaline pH-shift method affected the protein extraction efficiency from tilapia fillets. The procedure used ultrasound at 20 kHz with a short pulse cycle during the alkaline solubilization step of the process. Ultrasound significantly increased protein recovery at pH 10.5, compensating for the recovery achieved at pH 11.5 without ultrasound, making the process milder. Pezeshk et al. [10] found that the ultrasound-assisted pH-shift method similarly enhanced protein extraction from rainbow trout by-products. Different ultrasound powers and pH levels were tested, and the combined pH-shift-ultrasound treatment improved both solubilization and total protein yield compared to the pH-shift method alone. Furthermore, Pezeshk et al. [17] reported that ultrasound increased the yield of collagen recovered from tuna skin compared to conventional methods. In another study, Santschi et al. [33] investigated whether using ultrasound in combination with the classic pH shift method can mitigate a loss of protein extraction yield while decreasing the water ratio. Their findings showed that when the water ratio was reduced from the usual 1:6 to 1:3 for herring frames, ultrasound-assisted pH-shift processing successfully made up for yield loss. This technique didn't work with salmon heads, though. This disparity could be explained by variations in the two raw materials' composition, specifically in the amounts of sarcoplasmic and myofibrillar proteins. When the water ratio was reduced during pH-shift processing, ultrasound compensated for yield loss in herring frames, though it was ineffective with salmon heads. Ultrasound has also been widely targeted for the extraction of collagen and gelatin from fish by-products. The efficacy of ultrasonication in the extraction of acid-soluble collagen (ASC) and pepsin-soluble collagen (PSC) from golden carp skin was investigated by Moula Ali et al. [35]. The yield of ASC was greatly increased by applying ultrasound at 20 kHz with different amplitudes. Specifically, utilizing an 80 % amplitude in combination with varied amounts of pepsin resulted in greater yields of PSC. Furthermore, increased yields of both ASC and PSC were a result of longer ultrasonication periods. Shaik et al. [36] studied how ultrasound-assisted extraction affects collagen extraction from the skin of the Sharpnose stingray (*Dasyatis zugei*). The researchers found that this strategy has great promise for boosting collagen output while preserving the inherent properties of the collagen. In a second study, using an ultrasound bath at 60 °C for varying durations during gelatin extraction from bighead carp scales resulted in much higher extraction yield compared with a standard water bath method [37]. Gharib Heidari and Rezaei [38] and Gharib Heidari et al. [39] demonstrated that ultrasound-treated before treatment with rainbow trout pepsin and a commercial one significantly enhanced collagen extraction from yellowfin tuna skin. Petcharat et al. [40] discovered that prolonged ultrasonication led to higher yields regardless of amplitude. Among the tested amplitudes, 80 % produced the highest yield, attributed to the cavitation effect, which disrupts cell membranes and promotes collagen release. They concluded that an 80 % amplitude for 10 min was optimal for balancing yield and molecular integrity. In another study, 30 min of sonication resulted in the highest protein concentration during the extraction of *Coregonus peled* protamine using ultrasound-assisted acid extraction [41]. These variations in identified optimum conditions reveal a significant species, biomass, process and product specificity/dependency when employing ultrasound as assistant technology, which will define the optimum processing condition and needs to be identified for each individually.

Altogether, ultrasound-assisted extraction significantly enhances the water solubility of fish proteins by promoting structural alterations that facilitate protein solubilization. The high-energy cavitation generated during ultrasonication disrupts cellular structures, improves solvent penetration, and induces protein unfolding. These effects lead to the breaking of intra- and intermolecular cross-links, making fish proteins more soluble in aqueous extraction environments. Studies have demonstrated that ultrasound, particularly when combined with techniques such as the pH-shift method, increases the yield of alkali-soluble proteins from very complex fish by-products. The ability to extract high-quality, water-soluble proteins from fish by-products using ultrasound offers significant advantages over traditional extraction methods, including reduced use of solvents, shorter processing times, and the preservation of protein functionality, making it a promising approach for sustainable protein extraction in food applications.

## 5 The effect of ultrasound on the structural properties of fish proteins

Proteins are biological molecules with a hierarchical structure that contains primary, secondary, tertiary, and quaternary components. The primary structure determines the amino acid sequence of the polypeptide chain, while the secondary structure contains local folding configurations such as alpha-helices and beta-sheets. The tertiary structure is the three-dimensional arrangement of the complete protein molecule that is sustained by several interactions such as hydrogen bonds, hydrophobic forces, and disulfide bonds. The quaternary structure is formed by the assembly of several protein subunits into a functional complex [8].

Ultrasound may break down proteins into smaller peptides, causing structural changes at various levels, including secondary, and tertiary structures [43], whereas low-intensity ultrasound may disrupt weak noncovalent interactions, resulting in partial protein unfolding. Structure alterations (such as protein unfolding, denaturation, and refolding) have a significant impact on protein function. Considering the value of fish proteins as a dietary source, a thorough examination of these results is essential. The structural changes caused by ultrasound are highly dependent on specific processing circumstances such as power, time, and temperature, as well as the kind and biological features of the protein involved [21]. Several techniques are used to evaluate structural modifications in proteins, including circular dichroism (CD), Fourier transform infrared spectroscopy (FTIR), ultraviolet (UV) spectroscopy, intrinsic fluorescence spectrum analysis, X-ray diffraction (XRD), particle size, and scanning electron microscopy (SEM). These methods provide a thorough analysis of changes in protein folding, unfolding, and structural organization. Furthermore, other research has looked at the effects of ultrasound on fish protein structures and functionalities, which will be discussed in further detail in the coming sections as summarized in Table 2.

**Table 2:** Effect of ultrasound treatment and its combination with other treatments on fish proteins structure and functional properties.

Source	Method	Ultrasound conditions	Functional properties	Structure changes	Determinationmethods	References
Myofibrillar protein of *White Croaker*	Ultrasound	Frequency (kHz):20; Power (W): 500; Time (min):2, 4, 6, 8 and 10; Pulse on/off (s):3/3 [Optimal: Time → 10 min]	Solubility↑ Gel properties ↑	**Secondary structure:** α-helix ↓, β-sheet, β-turn random coil ↑ **Three-dimensional conformation:** tertiary and quaternary structure altered, positioning tryptophan in a polar environment. **Microstructure:** Protein particle size ↓ and uniform distribution ↑	FTIR UV Intrinsic fluorescence spectrum analysis Particle size	[42]
Myofibrillar proteins from *Coregonuspeled*	Ultrasound	Frequency (kHz):20; Power (W): 100, 150, 200 and 250; Time (min):3, 6, 9 and 12; Pulse on/off (s):2/2 [Optimal: Power → 200 W; Time → 9 min]	Solubility↑ Emulsifying properties↑	**Secondary structure:** α-helix ↓, β-sheet ↑, β-turn ↑ (initially at 150 W, then ↓ at 200 W), random coil initially ↑and then ↓. **Three-dimensional conformation:** tertiary and quaternary structure altered, positioning tryptophan in a polar environment. **Microstructure:** Protein particle size ↓ and uniform distribution ***↑***	FTIR Intrinsic fluorescence spectrum analysis Particle size	[61]
Protein of cod	Ultrasound	Frequency (kHz):20; Power (W): 200, 400, 600, 800 and 950; intensity (W/cm2): 75–83, 105–110, 131–138, 156–164 and 177–185; Time (min): 60; Pulse on/off (s):2/2 [Optimal: Power → 800 and 950 W]	Emulsifying properties↑	**Secondary structure:** α-helix ↓, β-sheet ↑, β-turn ↑ (initially at 150 W, then ↓ at 200 W), random coil initially ↑and then ↓. **Three-dimensional conformation:** tertiary and quaternary structure altered, positioning tryptophan in a polar environment. **Microstructure:** Protein particle size ↓ with uniform distribution ↑	CD Intrinsic fluorescence spectrum analysis Particle size	[52]
Protamine of *Coregonus peled*	Ultrasound-assisted acid extraction	Frequency (kHz):20; Power density (W/cm2): 9.10; Time (min): 10, 20, 30, 40, and 50 [Optimal: Time → 30 min]	Solubility**↑** Zone of inhibition **↑**	**Secondary structure:** α-helix and β-sheet initially ↑, then ↓ and β-turn initially ↓, then ↑; **Three-dimensional conformation:** Altered tertiary and quaternary structure, increased solvent exposure of hydrophobic groups. **Microstructure:** Protein particle size ↓ with structural disruption.	FTIR Intrinsic fluorescence spectrum analysis SEM	[41]
Myofibrillar protein of Asian sea bass meat	Ultrasound	Frequency (kHz): 20 ± 50; amplitude: 40 % and 60 %; Power (W): 750; Time (min): 5, 10, and 15; Pulse on/off (s):2/4 [Optimal: amplitude → 40 %; Time → 15 min]	Solubility↑ Emulsifying properties↑	**Secondary structure:** β-sheet and Random coil ↓, Hydrogen bonding ↑.	FTIR	[59]
Myofibrillar protein of Common carp	Ultrasound thawing	Frequency (kHz): 30 kHz; Power (W): 100, 300, and 500; Time (min): Ultrasound began until the center temperature of the sample rose to 4 ◦C [Optimal: Power → 300 W]	Gel properties↑ WHC↑	**Microstructure:** Ultrasound at medium power (300 W) improved the gel structure and particle size↓	Particle size SEM	[58]
Collagen of yellowfin skin	Pepsin + Ultrasound	Frequency (kHz):20; Power (W): 400; Time (min): 5, 10, 15, and 20; Pulse on/off (s):2/3 [Optimal: Time → 20 min]	Solubility↑ Emulsifying properties↑ Foaming properties↑ Gel properties↑ WHC↑	**Secondary structure:** minor shifts in amide peaks observed **Three-dimensional conformation:** minimal conformational changes observed.	FTIR UV	[39]
Collagen of yellowfin skin	Pepsin + Ultrasound	Frequency (kHz):20; Power (W): 400; Time (min): 5, 10, 15, and 20; Pulse on/off (s):2/3 [Optimal: Time → 20 min]	Solubility↑ Emulsifying properties↑ Foaming properties↑ Gel properties↑ WHC↑	**Secondary structure:** α-helix and β-turn ↓, β-sheet and random coil ↑ **Three-dimensional conformation:** Partial conformational changes observed	FTIR UV	[38]
Myofibrillar protein of Silver Carp	Ultrasound	Frequency (kHz):40; Power (W): 300; Time (min): 10, 20, and 30 [Optimal: Time → 30 min]	Solubility**↑** Gel properties**↑** WHC ns	**Secondary structure:** α-helix and β-sheet ↑, random coil ↓ **Three-dimensional conformation:** tertiary and quaternary structure altered, positioning tryptophan in a polar environment. **Microstructure:** Protein particle size ↓ and uniform distribution ↑, reducing voids in the gel matrix	CD FTIR UV Intrinsic fluorescence spectrum analysis Particle size SEM	[53]
Myofibrillar proteins of yellowstripe scad	NaCl + Ultrasound	Frequency (kHz):45; Power (W): 250; Time (min): 10; Pulse on/off (s): ns	Gel properties↑ WHC↑	−	−	[62]
Myofibrillar proteins of small yellow croaker	Ultrasound thawing	Frequency (kHz): 20, 40, 60 (depending on the treatment: MUT, DUT, TUT); Power (W): 250; Time (min): 10; Pulse on/off (s): ns; Water flow rate (rpm): 300 [Optimal: DUT (20 + 40 kHz)]	Gel properties↑	**Secondary structure:** α-helix ↓, β-sheet and random coil ↑ **Three-dimensional conformation:** tertiary and quaternary structure altered, positioning tryptophan in a polar environment. **Microstructure:** Protein particle size ↓ and uniform distribution ↑, reducing voids in the gel matrix	FTIR Intrinsic fluorescence spectrum analysis Particle size SEM	[48]
Myofibrillar protein of Common carp	Ultrasound thawing	Frequency (kHz): 30; Power (W): 100, 300 and 500; Time (min): 35.5–59.05; Pulse on/off (s): 30/30 [Optimal: Power → 300 W]	Solubility↑ Emulsifying properties↑ Thermal stability↑	**Secondary structure:** α-helix and β-turn↓, β-sheet and random coil ↑ **Three-dimensional conformation:** tertiary and quaternary structure altered, increased solvent exposure of tryptophan. **Microstructure:** Protein particle size ↓ and uniform distribution ***↑***	CD FTIR Intrinsic fluorescence spectrum analysis Particle size	[47]
Collagen of Sharpnose stingray skin	Acid and pepsin + Ultrasound	Frequency (kHz): 20; Power (W): 750; Time (min): 30; Pulse on/off (s): 5/5	Solubility (a pH range of 1–5) ↑	**Secondary structure and Microstructure:** No significant change between UASC and UPSC.	FTIR SEM	[36]
Gelatin of *Cyprinus carpio* L. scale	Ultrasound-assisted alkaline hydrolysis	Frequency (kHz): 20; Power (W): 360; Time (min): 20; Ultrasound Amplitude: 50.93 %; Pulse on/off (s): 2/2.	Solubility ↑	**Secondary structure:** α-helix ↓, β-sheet ↑ **Three-dimensional conformation:** Altered tertiary and quaternary structure, increased solvent exposure of hydrophobic groups. **Microstructure:** Protein particle size ↓ and uniform distribution ↑, structural integrity improved	FTIR Intrinsic fluorescence spectrum analysis Particle size XRD SEM	[50]
Protein of Hybrid sturgeon	Ultrasound + Enzymolysis	Frequency (kHz): 20; Power (W): 500; Time (min): 15, 30, and 45; Pulse on/off (s): 2/2 [Optimal: Time → 30 min]	Solubility ↑ Emulsifying properties↑ WHC ↑	−	−	[68]
Collagens from the skin of golden carp	Acid and pepsin + Ultrasound	Frequency (kHz): 20; Power (W): 750; Time (min): 10, 20, and 30; Amplitude: 20, 50, and 80 %; Pulse on/off (s): 5/5 [Optimal: Time → 30 min; Amplitude → 80 %]	−	**Secondary structure:** α-helix, β-turn, β-sheet; ns **Three-dimensional conformation:** Tertiary and quaternary structure largely retained.	CD FTIR	[35]
Myofibrillar proteins and hydrolysates of Golden threadfin bream	Ultrasound + Microwave	Frequency (kHz): 28; Power (W): 100, 200, 300, and 400; Time (min): 8 [Optimal: Power → 300 W]	Solubility ↑	**Secondary structure:** α-helix ↓, β-sheet ↑ (at high intensity), β-turn ↑ (at low intensity), random coil ↑ (moderate unfolding). **Three-dimensional conformation:** Protein unfolding ↑ (low intensity), aggregation ↑ (high intensity), hydrophobic interactions ↑, electrostatic interactions altered. **Microstructure:** Protein particle size ↓ (low intensity), aggregate formation ↑ (high intensity), uniform distribution ↑ (moderate intensity), structural rearrangement observed.	Raman spectroscopic analysis XRD Particle size SEM	[55]
Proteins isolated from rainbow troutby-products	pH-Shift + Ultrasound	Frequency (kHz): 20; Power (W): 100 and 400; intensity (W/cm2) 88 and 353; Time (min): 5, 10and 20; Pulse on/off (s): 2/3 [Optimal: Power → 400 W; Time → 10 min]	Solubility↑ Emulsifying properties↑ Foaming properties↑	**Secondary structure:** α-helix ↓, β-sheet ↑, β-turn ↑, random coil ↑. **Three-dimensional conformation:** Protein unfolding ↑, hydrophobic interactions ↑. **Microstructure:** Protein particle size ↓, aggregate formation ↑ (high power and time).	FTIR Intrinsic fluorescence spectrum analysis Particle size SEM	[10]
Collagen from tunaby-product	Acid + Ultrasound	Frequency (kHz): 20; Power (W): 300; Time (min): 5, 10, 15, 20and 25; Pulse on/off (s): 3/2 [Optimal: Time → 20 min]	Solubility↑ Emulsifying properties↑ WHC ↑	**Secondary structure:** ns **Three-dimensional conformation:** maintained tertiary and quaternary structures, enhanced hydrophobic interactions. **Microstructure:** Protein particle size ↓ and uniform distribution ↑, structural integrity improved	FTIR UV XRD Particle size SEM	[17]
Myofibrillar proteins from goldenthreadfin	Ultrasound-assisted tannicacid, quercetin and resveratrol	Power (W): 300; Time (min): 20; Pulse off (s): 2	Solubility↑ Emulsifying properties↑ Foaming properties↑	**Secondary structure:** α-helix ↓, β-sheet ↑, β-turn ↑, random coil ↑. **Three-dimensional conformation:** Protein unfolding ↑, hydrophobic interactions ↑.	CD FTIR UV Intrinsic fluorescence spectrum analysis	[46]
Myofibrillar protein of Japanese seerfish	Ultrasound −assisted acid	Frequency (kHz): 20; Power (W): 400; Time (min): 10; Pulse on/off (s): 2/2	Solubility↑ Gel properties↑	**Three-dimensional conformation:** Protein unfolding ↑, hydrophobic interactions ↑. **Microstructure:** Protein particle size ↓, structural rearrangement observed.	Intrinsic fluorescence spectrum analysis Particle size SEM	[57]
Protein hydrolysates from Atlantic mackerel	Ultrasound	Frequency (kHz): 20; Power (W): 300, 450, 600; Time (min): 10 [Optimal: Power → 450 W]	Solubility↑	**−**	−	[69]
Collagen from Albacore	Ultrasound	Frequency (kHz): 20; Power (W): 150, 300, 450, and 600; Time (min): 30; Pulse on/off (s): 2/2 [Optimal: Power → 150 W]	Gel properties↑	**Secondary structure:** Triple helix maintained **Three-dimensional conformation:** preserved tertiary structure and interchain spacing. **Microstructure:** wrinkled surface, cave-like morphology ↑, fiber looseness ↑, uniformity ↑	FTIR UV XRD SEM	[4]
Collagen from fish scales	Ultrasound + Natural deep eutectic solvents	Frequency (kHz): 20; Power (W): 450; Time (min): 20; Pulse on/off (s): 5/5	Solubility↑ Emulsifying properties↑ WHC↑	**Secondary structure:** Triple helix mostly retained. **Three-dimensional conformation:** Tertiary structure preserved with slight unfolding. **Microstructure:** porosity**↑**, roughness, and fiber loosening	FTIR XRD SEM	[60]
Nemipterus virgatus surimi	Ultrasound + Curdlan	Frequency (kHz): 40; Power (W): 150; Time (min): 20	Gel properties↑ WHC↑	**Secondary structure:** Shift from α-helix to random coil and β-turn. **Three-dimensional conformation:** Unfolding and protein cross-linking↑ **Microstructure:** Porosity↓ and more compact, dense network	FTIR SEM	[23]
Fish gelatin	Ultrasound + Phosphorylation	Power (W): 200; Time (min): 30, 60, 90, and 120 [Optimal: Time → 60 min]	Gel properties**↑**	**Secondary structure:** α-helix ↑ and random coil ↓, peak α-helix at UP60; α-helix/random coil ratio ↑ **Microstructure:** Porosity ↓, denser and more uniform gel network, improved gel strength and water retention	FTIR SEM	[70]
Silver carp surimi	Ultrasound + β-glucan	Frequency (kHz): 25; Intensity (W/cm2): 75.6; Time (min): 30	Gel properties**↑** WHC**↑**	**Microstructure:** Porosity ↓, denser and more uniform gel network, enhanced gel strength	SEM	[71]
Common carp myofibrillar protein	Ultrasound + Thawing	Frequency (kHz): 30; Power (W): 100, 300 and 500 [Optimal: Power → 300 W]	Gel properties**↑** WHC**↑**	**Three-dimensional conformation:** protein unfolding and hydrophobic interactions**↑** **Microstructure:** protein particle size↓ and observed structural rearrangement	Particle size SEM	[58]
Gelatin from Hilsha Scale	Ultrasound	Frequency (kHz): 20 and 130; Power (W): 200; Time (min): 60; Pulse on/off (s): 5/2	Emulsifying properties↑ Foaming properties↑ WHC**↑**	**Secondary structure:** Shift from α-helix to random coil **Three-dimensional conformation:** Unfolding ↑, intermolecular cross-linking ↓	FTIR UV	[9]

A commonly used technique for studying the secondary structure changes of proteins is CD [44]. The α-helix has a positive absorption band at 191–193 nm, followed by two negative peaks at 208–210 nm and 222 nm. The β-sheets are characterized by a positive band around 195–200 nm and a negative peak at 216–218 nm. Random coils exhibit a unique negative band in the range of 195–200 nm [45]. Wei et al. [46] reported that ultrasound pretreatment significantly altered the secondary structure of fish myofibrillar proteins by reducing α-helix content and increasing β-sheet structures, attributed to disruption of hydrogen bonds. Similarly, Sun et al. [47] observed that high-power ultrasound reduced helical structures, while lower intensities maintained more of the native α-helix. Wang et al. [48] also found that ultrasound decreased α-helical content and increased β-sheets. In contrast, Mola Ali et al. [49] reported no noticeable effect on the secondary structure of collagen, though ultrasound improved extraction efficiency by enhancing enzymatic cleavage. Furthermore, ultrasound-assisted alkaline treatment led to further shifts in protein structure, likely due to combined mechanical and electrostatic effects [50].

**Fig. 3:**
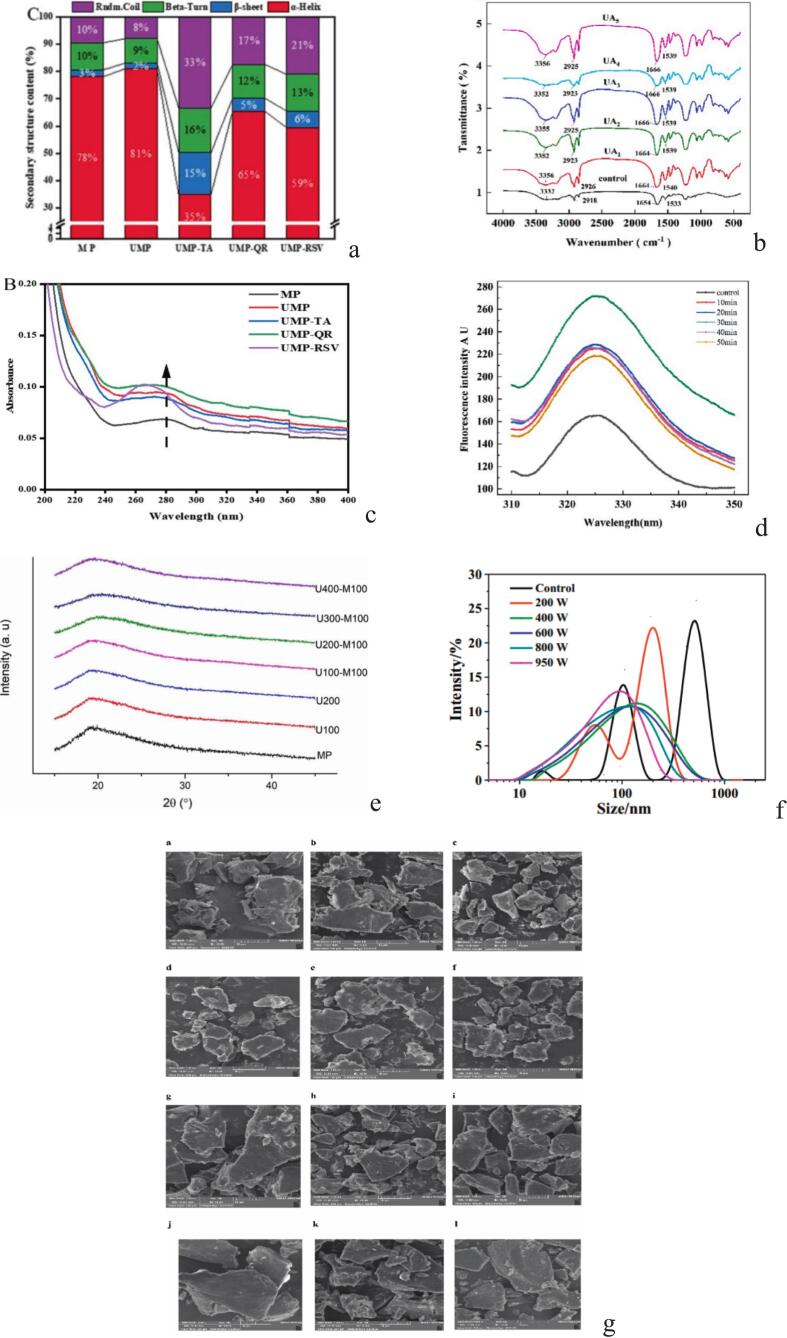
Structural changes of fish protein after ultrasound based on Circular dichroism (CD) spectra (a, adapted from Wei et al. [46]), Fourier transform infrared (FTIR) spectroscopy (b, adapted from Wang et al. [41]), UV spectroscopy (c adapted from Wei et al. [46]), fluorescence spectra (d, adapted from Wang et al. [41]), X-ray diffraction (XRD), (e, adapted from Li et al. [55]), particle size (f, adapted from Ma et al. [52]) and scanning electron microscope (SEM) (g, adapted from Pezeshk et al. [10]) techniques.

FTIR is an effective method for identifying protein structural changes through studying how different compounds absorb infrared light. Protein molecules might especially absorb certain infrared light wavelengths as a consequence of this mechanism, leading to molecular vibrations. The secondary structural elements of proteins, such as α-helices, β-sheets, β-turns, and random coils, may be identified more easily by analyzing the amide I band (1700–1600 cm^−1^) [45]. As shown in Fig. 3b, all *Coregonus peled* protamine samples exhibited absorption peaks in the 3600–3200 cm^−1^ range, corresponding to O–H and N–H stretching vibrations. Ultrasound shifted this peak from 3332 to 3356 cm^−1^, reflecting changes in intra- and intermolecular hydrogen bonding [41]. Similarly, Liu et al. [50] reported a shift in the amide B region from 2929 to 2811 cm^−1^, with increased peak intensity, suggesting enhanced hydrophobic interactions under ultrasound-assisted alkaline treatment. Ultrasound was also shown to disrupt α-helix content by weakening hydrogen bonds and increasing protein flexibility [42]. However, not all proteins responded similarly. Shaik et al. [36] observed that ultrasound had no major impact on the FTIR spectra of acid- and pepsin-soluble collagen, as amide band positions remained stable, preserving the triple-helical structure. In contrast, Pezeshk et al. [17] found that ultrasound could strengthen hydrogen bonding, shifting Amide A, though Amide III/CH_2_ ratios still indicated structural stability. These findings highlight how ultrasound alters protein interactions and secondary structure depending on protein type and treatment conditions, often improving functional properties via partial unfolding.

UV spectroscopy is an important method for examining protein structures by analyzing particular amino acid residues like tyrosine, phenylalanine, and tryptophan [36]. Several studies have shown that ultrasound treatment preserves the triple-helix structure of collagen. For example, Pezeshk et al. [17], Gharib Heidari & Rezaei [38], and Gharib et al. [39] observed that absorption peaks between 220–280 nm, especially around 232–235 nm, which are attributed to functional groups such as –COOH, CONH_2_, and C=O in collagen polypeptide chains. Also, Wei et al. [46] found that combining the ultrasound and polyphenols somewhat enhanced the UV absorption spectra of myofibrillar proteins, with a peak at 270 nm (Fig. 3c). This enhancement showed protein unfolding and chromophore exposure as a result of the change from a hydrophobic to a polar site. Furthermore, a red shift at 280 nm indicated a decrease in microenvironmental polarity near aromatic residues, which showed conformational changes. Similarly, Wang et al. [42] reported that ultrasound significantly affected the structure of myofibrillar proteins. Increased absorbance between 220–270 nm indicated a hyperchromic effect because of protein unfolding and the exposure of aromatic amino acids. A red shift at 220 nm indicated additional structural changes, like conjugated system extension and lower electron transition energy. These studies show ultrasound treatment improves the functional properties of proteins by inducing specific structural modifications.

The intrinsic fluorescence emission spectra are a good prognosticator of changes in the tertiary structure of protein. The tryptophan (Trp) emission peak is usually about 350 nm, while it is fully submerged in an aquatic environment. On the other hand, the emission peak shows a detectable blue shift, frequently occurring between 325 and 335 nm, when Trp is in a hydrophobic environment, like being placed in a protein or a membrane [51]. According to Wang et al. [41], ultrasound significantly influenced the intrinsic fluorescence intensity of Trp residues in *Coregonus peled* protamine (CPP), initially enhancing it due to unfolding and Trp exposure, followed by a decline with prolonged sonication as a result of protein refolding and reduced residue exposure. This dual-phase behavior indicates ultrasound's dynamic impact on protein structure. Ma et al. [52] observed a blue shift and increased fluorescence intensity in cod protein under ultrasound, suggesting rearrangements exposing Trp residues to a more hydrophobic environment, likely due to partial refolding or aggregation. Similarly, Wang et al. [42] reported a red shift in emission wavelength over time, reflecting structural unfolding and migration of Trp residues to polar environments. Xie et al. [53] confirmed that ultrasound combined with mild heat caused initial fluorescence reduction followed by a red shift, indicating progressive conformational changes. Pezeshk et al. [10] further supported this by showing a consistent decline in Trp fluorescence under increasing ultrasound power and time across pH levels, linked to aggregation and exposure of hydrophobic groups to aqueous surroundings.

XRD is a common technique for studying the crystal structure of proteins, especially to evaluate the effect of a treatment such as ultrasound [54]. Li et al. [55] reported that ultrasound at 200 W reduced the XRD peak at 2θ = 20°, indicating partial disruption of myofibrillar protein structure. Combined ultrasound–microwave treatment (U400-M100) further increased structural decomposition and amorphous content, enhancing solubility (Fig. 3e). Similarly, Liu et al. [50] observed peaks at 2θ = 6.4° and 20.8° in fish scale gelatin. Although gelatin lacks collagen’s native triple helix, these peaks may reflect residual structural order. Crystallinity rose from 3.02 % (control) to 8.72 % (ultrasound), and 12.54 % with additional alkaline treatment, attributed to cavitation and the disruption of non-covalent bonds. Pezeshk et al. [17] also found XRD peaks at 2θ ≈ 7.5 and 21.2 in sonicated collagen, corresponding to triple helical structure, with interchain distances of 11.6 and 4.1 Å. Notably, the collagen’s natural structure remained intact despite treatment.

According to Qian et al. [56], particle size strongly affects protein functionality, including solubility, structure, and aggregation. Ma et al. [52] reported that ultrasound reduced cod protein aggregates. At 200 W, the control's three size peaks (16.7, 105.7, and 530.3 nm) shifted to two peaks (60.0 and 189.7 nm), confirmed by DLS. Higher intensities (400–950 W) led to more uniform particles between 92–155 nm (Fig. 3f). Similarly, Pan et al. [57] found that ultrasound reduced fish protein size from 1100 nm to 230–270 nm due to cavitation breaking aggregates. Sun et al. [58] also observed size reduction at 300 W, but at 100 or 500 W, particle size increased, likely due to insoluble aggregate formation. These findings highlight the critical role of ultrasound intensity, as improper levels may produce adverse effects.

SEM technique is widely used to examine the appearance and surface morphology of proteins, especially to investigate disintegration or aggregation. Pezeshk et al. [10] reported that ultrasound treatment significantly altered protein aggregates. In an alkaline environment, sonication at 400 W reduced aggregate size and produced more irregular structures compared to lower power levels, indicating that higher intensity leads to smaller particles. However, under acidic conditions, the same intensity promoted aggregation, likely due to over-processing and protein damage. These morphological changes influenced functional properties such as emulsifying and foaming capacities (Fig. 3g). Similarly, ultrasound at 200 W reduced large MP aggregates to smaller, more uniform fragments. Combined ultrasound-microwave treatments further decreased aggregation, resulting in more homogeneous structures [55]. Shaik et al. [36] found that ultrasound-assisted extraction altered the surface morphology of collagen from Sharpnose stingray skin, creating porous and more regular structures compared to the coarse state of the unsonicated samples. Pezeshk et al. [17] also stated that ultrasound caused physical changes in the collagen structure and a more porous and regular network pattern was created. When the ultrasound time was increased to 25 min, the length of the fibrils and their structure changed, which was attributed to the cavitation effect, resulting in modifications to collagen properties.

In summary, ultrasound profoundly alters the structural properties and dynamics of fish proteins by disrupting hydrogen bonds, modifying secondary and tertiary structures, and promoting protein unfolding and exposure of functional groups. These structural effects are highly dependent on the sonication parameters—particularly amplitude, intensity, frequency, treatment time, and temperature. Studies reviewed in this section consistently show that increased ultrasonic power or extended treatment durations lead to significant unfolding or even aggregation of proteins, driven largely by cavitation forces and localized thermal effects. Prolonged ultrasonication may also generate reactive oxygen species, causing oxidative damage to amino acid residues and compromising structural stability. Techniques such as CD, FTIR, UV, fluorescence spectroscopy, and XRD collectively demonstrate reductions in α-helix content, increases in β-sheet structures, shifts in vibrational and emission peaks, and changes in crystallinity, altogether reflecting a transformation toward more flexible and reactive protein conformations. However, when the ultrasound conditions exceed optimal thresholds, protein refolding or aggregation may occur, reducing flexibility and functional potential. Therefore, precise tuning of ultrasound parameters is essential to balance beneficial structural disruption with the risk of excessive denaturation. Since different fish protein types (e.g., collagen, myofibrillar, sarcoplasmic) respond variably to ultrasonication, further research is warranted to tailor ultrasound regimes to the specific characteristics of each protein type.

## 6 The effect of ultrasound on the functional properties of fish proteins

**Fig. 4:**
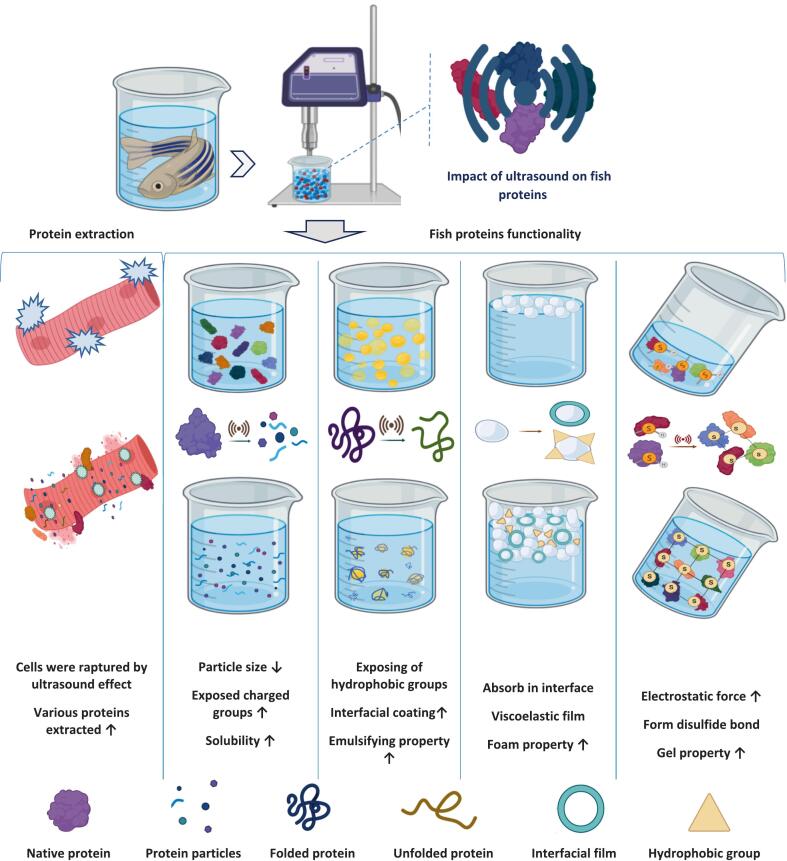
Schematic overview of the impact of commonly applied ultrasound technology on fish proteins' extraction and structure function dynamics.

The functional properties of fish proteins are mainly related to their wetting properties and surface properties, which are explained by protein-water, protein-fat and protein–protein interactions, including solubility, emulsification, foaming and gelling properties. An overview of the use of ultrasound in the extraction and altered functioning characteristics of fish protein is shown in Fig. 4.

### 6.1 Solubility in water

**Fig. 5:**
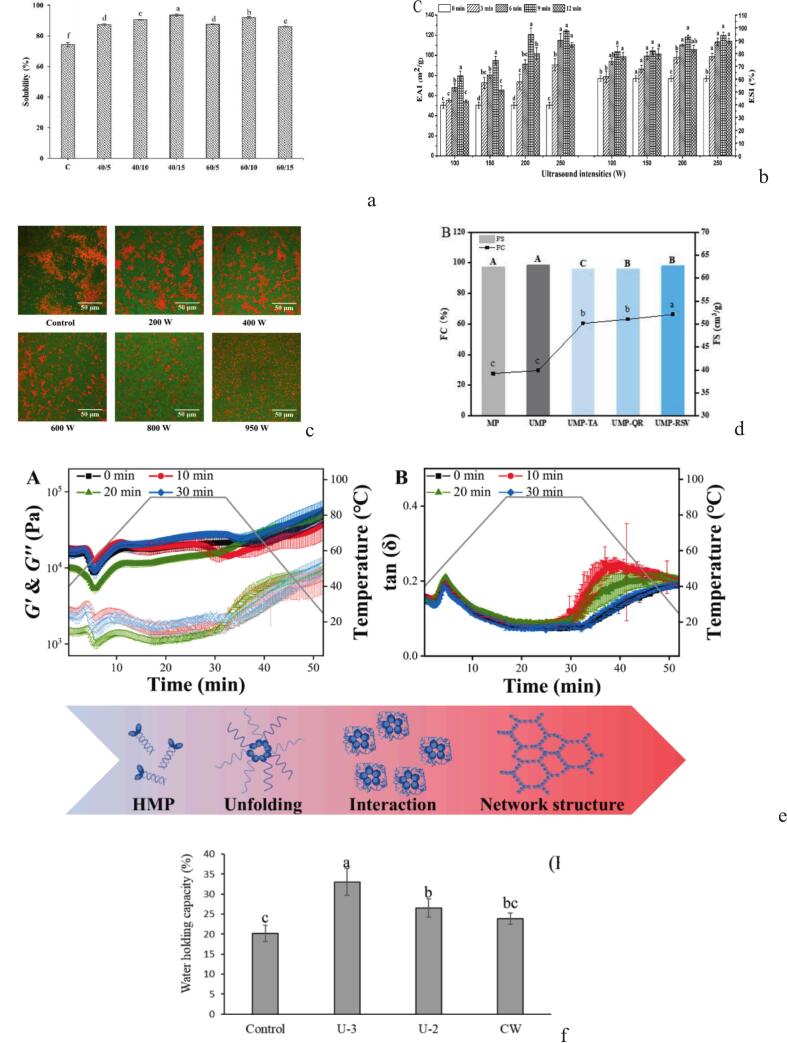
Functional changes of fish protein after ultrasound treatments, solubility (a, adapted from Rajaskaran et al. [59]), emulsify (b & c, Deng et al. [61] & Ma et al. [52], respectively), foaming (d, adapted from Wei et al. [46]), gelling (e, adapted from Xie et al. [53]), and WHC (f, adapted from Sinthusamran et al. [62]) properties.

The structural composition, type of amino acids and intermolecular interactions are important determinants of fish protein solubility. These proteins usually contain hydrophobic amino acids such as leucine, phenylalanine and valine, which tend to aggregate in water and are less soluble. Structural changes in proteins can increase their solubility in water by exposing thiol groups and hydrophobic phenyl groups [10]. Rajaskaran et al. [59] showed that ultrasound treatment, especially if performed for 15 min at 40 % amplitude, improved the solubility of fish muscle protein (FMP). As the treatment process continued, the structure of the proteins changed and their internal hydrophilic groups became more exposed, which increased solubility. On the other hand, some bonds were broken and the protein size became smaller. This was due to physical stresses such as mechanical shear, shock waves and cavitation, which improve water-protein interactions. However, when the treatment time increased, for example, above 10 min and with an amplitude of 60 %, the result was the opposite: the protein was denatured, and hydrophobic bonds increased. Thus, the protein reassembled and the water-protein interactions were reduced (Fig. 5a). Similarly, Sun et al. [47] found that at an intensity of 300 W, the solubility of fish protein improved because aggregation and denaturation were less. However, when the power exceeded 500 W, the solubility decreased, indicating that overtreatment has the opposite effect. Furthermore, Li et al. [55] found that ultrasound treatment at 100 W and 200 W greatly improved fish muscle protein solubility by breaking down protein aggregates into smaller particles with more surface area and exposed polar residues. However, more than 60 % of muscle proteins remained insoluble after 200 W treatment at pH 7. Remarkably, the combination of microwave-ultrasound treatment (U300-M100) resulted in solubility of 83–100 % across pH 2–10, indicating its ability to change the protein structure and increase its water-binding capacity. Pezeshk et al. [17] also reported that collagen became more soluble at all pHs after ultrasound treatment. This was likely due to reduced cross-linking and weaker intermolecular interactions, as well as smaller particles. All of these make the protein more easily soluble in water. Wang et al. [42] also showed that ultrasound treatment increased fish protein solubility. For example, after 4 min of treatment, the solubility of myofibrillar proteins increased from 61.35 % to 88.28 % (P < 0.05). This increase was due to the cavitation and turbulence effects of ultrasound, but after 6–10 min, no significant difference was observed.

Overall, ultrasound treatment has been consistently shown to enhance the water solubility of fish proteins through structural modifications such as unfolding, particle size reduction, and the exposure of hydrophilic and polar groups. These changes are primarily driven by mechanical effects including cavitation, shear forces, and turbulence, which disrupt protein aggregates and promote hydration. Moderate ultrasound conditions have proven particularly effective, increasing solubility across different fish protein types and pH ranges. However, excessive treatment, either in terms of power, duration, or amplitude, can reverse these benefits by inducing protein denaturation, increasing hydrophobic interactions, and leading to reaggregation, thereby reducing solubility. Therefore, the optimization of ultrasound parameters is critical to maximizing solubility improvements without compromising protein integrity. Future work should focus on tailoring ultrasound protocols to specific protein structures and processing goals, particularly in multifunctional food systems.

### 6.2 Emulsifying properties

An emulsion is a colloidal system formed by dispersing two or more immiscible liquids, typically oil and water. Proteins play a central role in stabilizing these systems by adsorbing at the oil–water interface and forming a viscoelastic interfacial film that prevents phase separation. Their amphiphilic nature possessing both hydrophilic and hydrophobic regions enables proteins to reduce interfacial tension and enhance emulsion stability. Given the intrinsic instability of emulsions due to droplet aggregation, coalescence, or creaming, the presence of proteins is crucial for maintaining structural integrity over time [11]. To evaluate the emulsification properties, the emulsification activity index (EAI) and the emulsion stability index (ESI) are commonly used. EAI indicates the ability of the protein to adsorb at the interface, while ESI refers to the degree of stability of the adsorbed protein layer on the surface of the emulsion droplets [60]. Ultrasound improves the emulsification properties of fish proteins in water-in-oil emulsions through several mechanisms. Ultrasound induces structural changes in the protein and exposes charged groups that increase the electrostatic repulsion between particles. As a result, droplet aggregation is prevented and a more stable and uniform emulsion is formed. Ultrasound-induced cavitation breaks large protein molecules into smaller peptides. These peptides are more easily adsorbed at the interface and stabilize the droplets. Ultrasound also reduces the particle size during the homogenization process and improves the emulsification efficiency by increasing the surface hydrophobicity of proteins [8]. Ultrasound treatment has shown varied effects on the emulsification properties of myofibrillar proteins, depending on processing conditions. Several studies reported that moderate ultrasound intensities (200–950 W) and durations (≤15 min) improved emulsifying activity index (EAI) and emulsion stability index (ESI), primarily due to cavitation-induced unfolding, increased surface hydrophobicity, reduced particle size, and enhanced interfacial adsorption. Deng et al. [61], Ma et al. [52], and Pezeshk et al. [10] all observed enhanced emulsification under optimized conditions, while CLSM images confirmed finer, more stable structures. Conversely, excessive ultrasound exposure or high-intensity treatments, especially in acidic conditions, led to protein aggregation, over-denaturation, and oxidation, ultimately reducing emulsification performance (Sun et al. [47]; Pezeshk et al. [10]; Gharib Heydari & Rezaei [38]). These findings highlight the importance of balancing ultrasound parameters to maximize emulsifying efficiency without compromising protein structure.

Overall, the results of existing studies emphasize the importance of optimally adjusting the ultrasound parameters to enhance the emulsification efficiency. Although ultrasound can effectively improve the emulsifying properties of fish proteins by modulating their structural and interfacial characteristics. Through cavitation-induced shear forces, ultrasound reduces protein particle size, increases solubility, and exposes hydrophobic and charged residues that promote stronger adsorption at the oil–water interface smaller droplet sizes, and reduced creaming and aggregation. However, its excessive use can lead to structural degradation, aggregation, and reduced emulsion quality. Therefore, understanding the boundary between structural improvement and destruction is essential for the proper tuning of fish protein emulsifying properties. Optimizing ultrasound conditions is critical to harness its full potential in improving the emulsifying functionality of fish-derived proteins, particularly for applications in structured or processed foods where stable emulsions are essential.

### 6.3 Foaming properties

The foaming properties of fish proteins are primarily due to their ability to form a flexible surface layer; a layer that can trap air and retain moisture. This combination allows for the formation of stable foams. Interestingly, ultrasound can considerably enhance these properties. Using the shear forces generated by cavitation, the protein structure is opened, fragmented, and more hydrophobic parts appear on the surface — all of which contribute to better foaming [8]. For example, Wei et al. [46] demonstrated that ultrasound induces protein unfolding, facilitating its adsorption at the air–water interface, which enhances foam formation and stability (Fig. 5d). Pezeshk et al. [10] showed that ultrasound treatment significantly increased the foaming capacity (FC) and foam stability (FS) of rainbow trout proteins, especially under alkaline conditions. Due to the unfolding of the protein structure, increased solubility and smaller particles. Another study by Gharib Heydari and Rezaei [38] showed the same results for collagen, however, they showed that when the treatment time was increased, FC and FS decreased. This was also due to the decrease in the concentration of active proteins. Although ultrasound can be a useful tool to improve foaming, its intensity and duration must be properly adjusted; otherwise, the result may be the opposite.

### 6.4 Gel-forming and textural properties

The gel-forming ability of fish proteins is a critical functional property that directly influences their applicability across a wide range of food products, particularly in terms of texture and structural integrity. A protein gel is a three-dimensional network formed by the aggregation of unfolded protein molecules, which trap water and other components, resulting in a semi-solid structure. Gel-forming ability refers to the capacity of proteins to undergo denaturation and subsequent aggregation under specific conditions, leading to the development of cohesive and elastic matrices stabilized with hydrogen, disulfide and hydrophobic bonds. This characteristic is especially vital in surimi-based products, where the formation of stable, elastic gels defines both product quality and consumer acceptability. Ultrasound treatment has shown promise in enhancing the gelling properties of fish proteins by promoting the formation of disulfide bonds and strengthening interprotein interactions through the exposure of sulfhydryl groups and hydrophobic regions. Additionally, the mechanical effects of cavitation help break down large protein aggregates into smaller, more uniformly distributed particles, resulting in a denser, more compact gel network with improved mechanical strength. These changes in protein conformation and interaction patterns influence key textural attributes such as hardness, cohesiveness, and elasticity, which are critical for consumer perception and processing performance. Furthermore, such structural improvements can reduce cooking loss and improve mouthfeel and overall acceptability of fish-based products. Xie et al. [53] reported that heat treatment combined with ultrasound significantly improved the gelling properties of silver carp surimi. This could be due to the unfolding of the protein structure, increased solubility, and strengthening of intermolecular crosslinks. The result was a smoother, more elastic, and stronger gel, with more disulfide bonds and stronger hydrophobic interactions. Rheological analysis (Fig. 5e) also confirmed this trend with a decreased phase angle (δ) and increased storage modulus (G') in gels made of ultrasound treated samples. Sintosamran et al. [62] also found that ultrasound-assisted washing enhances the gelation properties of surimi by increasing breaking strength and overall gel strength. The treatment facilitated better dispersion of sarcoplasmic proteins, lipids, and pigments, which in turn promoted stronger intermolecular interactions and more effective gel network formation. Moreover, ultrasound-induced cavitation increased myosin aggregation, contributing to a more uniform and elastic final gel structure. Wang et al. [48] found that ultrasound treatment was also reported to alter fish protein structure, reduce particle size, and enhance uniformity, leading to a more coherent final gel network. Pan et al. [57] also reported that low-intensity ultrasound treatment positively influenced protein functionality by promoting protein unfolding, increasing the exposure of sulfhydryl (–SH) and amino (–NH_2_) groups, and facilitating cross-linking during heating. In contrast, high-intensity treatment led to excessive protein aggregation, limiting available physical space and ultimately reducing gelation capacity. However, moderate ultrasound doses induced optimal structural modifications without triggering excessive aggregation, resulting in the highest gelation potential. Finally, Santschi et al. [33] showed that ultrasound treatment during the protein extraction from salmon head (SH) and herring frame (HF) using the pH-shift method did not significantly affect the viscoelastic properties such as G' and G'' of the proteins, especially in HF. This is important, as it suggests that factors such as the water-to-protein ratio and the composition of the raw materials may have a greater effect than ultrasound during the protein extraction.

Altogether, existing knowledge proposes that ultrasound treatment, particularly at low to moderate intensities, can significantly enhance the gel-forming ability of fish proteins by improving water solubility, promoting protein unfolding, and facilitating the formation of intermolecular interactions such as disulfide bonds and hydrophobic associations. While moderate disulfide bonding strengthens the gel network by stabilizing crosslinks, excessive bonding may rigidify the structure and reduce flexibility, ultimately impairing gel elasticity and water-holding capacity. Similarly, mild aggregation can support network formation, but uncontrolled aggregation typically reduces solubility and disrupts gel uniformity [62]. The mechanical action of ultrasound, including cavitation and shear forces, contributes to the breakdown of large protein aggregates and the exposure of functional groups, resulting in better solubility and a more uniform protein dispersion. These changes support stronger and more elastic gel network formation, as evidenced by improved rheological properties and textural attributes. However, excessively high ultrasound intensities may lead to over-aggregation, reduced solubility, and impaired gelation due to restricted molecular mobility. Additionally, variations in raw material composition and process parameters—such as pH, water-to-protein ratio, and washing steps—can modulate the final outcome. Overall, the ability of ultrasound to tailor the gelling behavior of fish proteins holds great promise for developing high-quality products and integrating these proteins into more complex and multifunctional food systems, such as structured seafood analogues and hybrid foods.

### 6.5 Water holding capacity

Water holding capacity (WHC) refers to a protein's ability to retain water molecules, and it is an important measure for determining water retention in protein gels as well as structural stability. WHC is affected by a variety of variables, including ionic strength and protein content. Enhancing the gel matrix improves its ability to absorb and hold water, improving WHC [63]. An efficient technique for raising the WHC of fish proteins is ultrasound. According to Sinthusamran et al. [62], ultrasound significantly increased the water-holding capacity (WHC) of fish proteins (Fig. 5F). This enhancement was attributed to structural modifications induced by ultrasound, including protein unfolding and the strengthening of intermolecular interactions—such as hydrogen bonding and hydrophobic interactions—associated with a reduction in α-helix content and an increase in β-sheet structures. These conformational changes improved protein flexibility and water retention. Additionally, ultrasound-assisted washing contributed to higher WHC by facilitating the removal of sarcoplasmic proteins and enhancing the exposure of myofibrillar proteins to water, thereby promoting more effective water binding. Similarly, Pezeshk et al. [17] demonstrated that by exposing hydrophilic and charged groups, ultrasound treatment greatly increased WHC and encouraged increased water retention. In contrast, Santschi et al. [33] found that ultrasound had no discernible impact (p > 0.05) on the WHC of protein gels produced from herring frames. This lack of effect was in line with the fact that there were no changes in gel stiffness, indicating that neither the gel network nor protein–protein interactions were altered by ultrasound. Rather than ultrasound treatment, WHC seemed to be largely influenced by protein composition, specifically the amount of myosin heavy chain. Generally speaking, this disparity may result from variances in the processing circumstances, the particular ultrasound parameters used, or the protein composition. This emphasizes the need for more investigation to clarify the variables influencing these disparate results.

Taken together, current findings confirm that the effects of ultrasound on fish protein functionality are tightly linked to its structural modifications, which are, in turn, highly dependent on the processing parameters. Amplitude and intensity primarily govern the extent of unfolding or aggregation, while frequency and sonication time influence dispersion and interfacial behavior. Temperature modulates both the efficiency of cavitation and the risk of protein denaturation. Therefore, achieving optimal functional outcomes—such as improved solubility, emulsification, or gelation—requires fine-tuning these variables based on the desired structure–function relationships.

## 7 Challenges and limitations of Ultrasound-Assisted extraction in fish protein processing

One of the major obstacles to the broader adoption of ultrasound-assisted extraction technology is the absence of standardized methodologies and clearly defined process parameters. Consistent documentation of key factors such as energy input, probe specifications, and sample handling conditions would greatly enhance the reliability of results and support more accurate techno-economic assessments for potential industrial applications. Although ultrasound has shown significant promise at the laboratory scale, its successful transition to industrial application remains constrained by several technical, methodological, and process-related limitations [21]. One key concern is the impact of high-power ultrasonication on protein structure. Depending on the ultrasound intensity and treatment duration, cavitation effects may induce partial unfolding or aggregation of fish proteins, which in turn can alter solubility, emulsification, foaming, and other functional properties. Additionally, localized temperature rise during prolonged sonication and the generation of reactive oxygen species can contribute to protein oxidation, possibly compromising the nutritional quality or bioactivity of the extracted proteins. Moreover, high-intensity ultrasound may impact protein digestibility by inducing structural changes that affect enzymatic breakdown. These modifications, along with possible oxidation of bioactive compounds, can influence the nutritional quality of the extracted fish proteins. Such changes in bioactive compounds may have both beneficial and adverse effects on the functional and nutritional properties of the extracted proteins [11]. Scalability is another important issue. Ultrasound systems often suffer from limited penetration depth and non-uniform energy distribution, especially in viscous or complex fish matrices. Industrial-scale systems may also encounter energy losses due to attenuation, equipment degradation, or cavitation inefficiencies across large sample volumes. These factors complicate process control and may require the integration of auxiliary mixing or pre-treatment steps to achieve homogeneous treatment. Moreover, the lack of consensus on optimal ultrasonic parameters (e.g., frequency, amplitude, time, and temperature) for different fish species and product targets poses a barrier to standardization and broad application. As noted by several authors, ultrasound may yield both beneficial and adverse effects depending on matrix composition, desired functionality, and downstream use [20]. Despite these limitations, ultrasound remains a valuable, tunable, and energy-efficient technology. With proper optimization and process monitoring, many of the associated drawbacks can be mitigated. Future research should focus on the development of standardized protocols, system-level integration strategies, and real-time control tools to improve reproducibility and unlock the full potential of ultrasound in the sustainable processing of fish proteins.

## 8 Conclusions and Future Perspectives

Ultrasound technology has emerged as a transformative tool in fish protein processing, offering significant advantages in both extraction efficiency and structure–function modulation. Ultrasound, particularly when combined with techniques such as the pH-shift method, increases the ability to extract high-quality, water-soluble proteins from fish by-products. Using ultrasound offers significant advantages over traditional extraction methods, including reduced use of solvents, shorter processing times, and the preservation of protein functionality, making it a promising approach for sustainable protein extraction in food applications. By leveraging high-intensity ultrasound effects, primarily cavitation, shear forces, and turbulence, this technique facilitates the disruption of cellular structures, unfolding of proteins, and exposure of functional groups. These mechanisms contribute to enhanced water solubility, improved emulsifying capacity, and superior gel-forming ability across a variety of fish protein types, including myofibrillar, sarcoplasmic, and collagen proteins. Moderate ultrasound treatments have consistently demonstrated the ability to improve functional properties while preserving protein integrity, making them highly suitable for applications in multifunctional food systems such as emulsions, gels, and hybrid seafood analogues. However, the efficacy of ultrasound is highly dependent on precise control of operational parameters, power, frequency, amplitude, and duration. Exceeding optimal thresholds can lead to protein degradation, reaggregation, or loss of desirable functionalities. Therefore, a nuanced understanding of protein-specific responses and process conditions is essential for maximizing the benefits of ultrasonication.

A number of challenges need to be addressed to effectively incorporate ultrasound into industrial processes. Process complexity, variability of repeatability, and limitations of large-scale equipment are among the factors that need to be considered when using ultrasound. To facilitate industrial adoption, more advanced ultrasound systems with greater control, as well as the integration of ultrasound with other processing technologies, may increase its efficiency in fish protein-related industries. Given these issues, it seems worthwhile to focus future studies on optimizing ultrasound processing for large-scale applications.

Future research should also focus on developing tailored ultrasound protocols that are compatible with diverse fish protein matrices and targeted food applications. Additionally, combining ultrasound with complementary techniques such as pH-shift processing or enzymatic treatments may further enhance its potential.

Looking ahead, ultrasound offers promising potential as a precision tool to fine-tune the structure of fish proteins, enabling the engineering of tailored functionalities that enhance their compatibility with complex food processing techniques such as high-moisture extrusion and 3D printing. Furthermore, such structural modulation may support the design of hybrid foods by improving the integration of fish-derived proteins with emerging protein sources, including plant, microbial, and cultured proteins, fostering the development of next-generation sustainable and functional food products.

## Data availability

Data will be made available on request.

## CRediT authorship contribution statement

**Samaneh Pezeshk:** Writing – original draft, Supervision, Conceptualization. **Mehdi Abdollahi:** Writing – review & editing, Supervision.

## Declaration of competing interest

The authors declare that they have no known competing financial interests or personal relationships that could have appeared to influence the work reported in this paper.
